# Association of Paternal BMI and Diet During Pregnancy with Offspring Birth Measures: The STEPS Study

**DOI:** 10.3390/nu17050866

**Published:** 2025-02-28

**Authors:** Michelle L. Kearns, Mirkka Lahdenperä, Laura Galante, Samuli Rautava, Hanna Lagström, Clare M. Reynolds

**Affiliations:** 1 School of Public Health, Physiotherapy and Sports Science, University College Dublin, Belfield, D04 V2P1 Dublin, Ireland; michelle.kearns1@ucdconnect.ie; 2 Department of Biology, University of Turku, 20014 Turku, Finland; mirkka.lahdenpera@utu.fi; 3 Medical School, Swansea University, Swansea SA2 8PP, UK; laura.galante@swansea.ac.uk; 4 Department of Pediatrics, University of Helsinki, 00100 Helsinki, Finland; samrau@utu.fi; 5 New Children’s Hospital, Helsinki University Hospital, 00029 Helsinki, Finland; 6 Centre for Population Health Research, University of Turku and Turku University Hospital, 20014 Turku, Finland; 7 Department of Public Health, University of Turku and Turku University Hospital, 20014 Turku, Finland

**Keywords:** diet quality, birthweight, paternal influences

## Abstract

Background/Objectives: Maternal Body Mass Index (BMI), diet quality, and their associated effects on offspring birth measures are well-established. Emerging evidence, largely from animal studies, has indicated paternal factors can influence offspring birth outcomes. However, this effect is poorly understood in humans. Our aim was to examine the association between paternal BMI and diet quality score and offspring birth measures. Methods: Participants from the STEPS (Steps to the healthy development) Study in Southwest Finland were recruited during the first trimester of pregnancy or after delivery. A total of 1586 fathers and their children were included for BMI analysis, and 208 fathers and their children were included for dietary analyses. Paternal BMI was calculated using self-reported weight and height at recruitment, and dietary behaviour was assessed using the Index of Diet Quality (IDQ) at 30 weeks’ gestation. Offspring birth weight and length z-scores were calculated using the recently published references specific to the Finnish population. Generalized linear model analyses were carried out to determine associations between paternal factors and offspring z-scores. Results: The mean paternal BMI was 26 (SD ± 3.5). Over half of the fathers were classed as having an unhealthy diet, classified as poor in adhering to nutrition recommendations including higher intakes of saturated fatty acids, and inadequate intakes of protein, saccharose, fibre, vitamins, and minerals. Paternal BMI was not significantly associated with offspring birth weight (β = 0.00 *p* = 0.884) or birth length (β = 0.00, *p* = 0.774) z-scores when adjusted for maternal and other paternal and parental factors. Paternal diet quality score was not associated with offspring birth weight (β = −0.01, *p* = 0.515) or birth length (β = 0.07 *p* = 0.291) z-scores. Conclusions: This study shows paternal BMI or diet quality at 30 weeks’ gestation does not significantly impact offspring birth measures. Given the known impact of nutrition on epigenetics, examining the potential influence of paternal factors at conception on offspring growth is of major importance and should be included in future studies.

## 1. Introduction

Obesity and type 2 diabetes mellitus rates are rising globally, with a prediction that by 2050, half a billion people will be type 2 diabetic, and may suffer from disease-related complications such as kidney and heart disease and premature mortality [1]. Fetal programming is now recognised as a contributing factor to this rise in obesity and Type 2 Diabetes Mellitus (T2DM) globally. Therefore, understanding these processes may help to tackle this global disease burden.

Birth weight and birth length, along with gestational age, are associated with immediate adverse outcomes such as perinatal morbidity and mortality [2]. Associations were also drawn between birth weight and cardiometabolic disease in later life [3,4,5]. In addition, birth length independent of birth weight is also related to disease incidence in later life, including coronary heart disease (CHD) [6]. Fetal growth during gestation depends on numerous factors, including hormonal regulation, maternal eating habits during pregnancy, as well as nutrient transfer capacity in the placenta to fuel fetal growth [7]. Insufficient nutrition during critical windows of fetal development can affect the development of organs and tissues, which can lead to permanent metabolic changes later in life [8].

Within the field of developmental origins of health and disease (DOHaD), there has been substantial focus on maternal health during pregnancy and offspring outcomes. This is unsurprising due to the influence of the maternal environment, particularly the supply of nutrients from the placenta to the fetus. A systematic review of epidemiological studies on maternal dietary patterns concluded that adherence to unhealthy dietary patterns was associated with low birth weight [9], while a systematic review by Heslehurst et al., found a 264% increase in the odds of child obesity when mothers have obesity before conception [10]. In addition to the well-known maternal influence, there is now emerging evidence of paternal contributing factors to offspring health, including paternal obesity and dietary patterns [11,12,13,14].

Most of the research examining paternal-offspring effects has been carried out in animal models. Male mice fed a high-fat diet (HFD) display increased sperm DNA fragmentation [15], impaired embryo development [16], and offspring adverse effects, including glucose intolerance and defective insulin secretion at 6 weeks old [17]. Furthermore, there is evidence that paternal diet may affect postnatal and long-term body weight in the offspring. Paternal pre-conception HFD resulted in decreased early postnatal body weight in mouse offspring [18]. In addition, rat offspring had increased body weight in later life in response to paternal HFD [17]. These findings have been reflected in limited human studies, with increased paternal BMI associated with increased offspring BMI and increased birth weight [19].

There is a paucity in the literature investigating the significant impact of paternal weight or diet quality around the time of conception on offspring birth weight or other measures in humans [20,21]. In addition, few studies have focused on parental influences on newborn’s birth length. Previous studies have reported maternal factors including smoking and short gestational length significantly decreased a child’s birth length [22]. However, few have looked at paternal influencing factors. Birth length z-score alone may be linked to health risks in infants, including bronchopulmonary dysplasia [23], and lower birth length was associated with overweight and obese early-adolescent boys [24].

To address the gap in knowledge of paternal influence on offspring birth outcomes, this study used data from the STEPS study, a Finnish cohort of newborn babies and their mothers and fathers, which reported data including paternal BMI and diet quality during gestation. This cohort was chosen due to the extensive paternal data collected, in addition to offspring and maternal data, helping to address the gap in human studies. We hypothesize that paternal BMI and diet quality impacts offspring birth measures. 

## 2. Materials and Methods

### 2.1. Study Participants

Participants included in this study were recruited to the Finnish longitudinal “Steps to Healthy Development of Children” (STEPS) study, which has been previously described [25]. Briefly, all Finnish- and Swedish-speaking mothers, who delivered a living child between 1 January 2008 and 31 April 2010 in the Hospital District of Southwest Finland, formed the cohort population (in total, 9811 mothers and their 9936 children). Altogether, 1797 mothers and 1658 fathers, and 1805 neonates (including twins) volunteered as participants for the intensive follow-up group of the STEPS study. For the present study, children born from singleton pregnancies, and where BMI and/or diet quality information was available from fathers, were included (*n* = 1640). In this study, we included fathers who had BMI (*n* = 1586) or diet (*n* = 208) information and their offspring.

The study was approved by the Ethics Committee of the Hospital District of Southwest Finland in February 2007. The parents gave their written informed consent for the study. The legal basis for processing of personal data is public interest and scientific research (EU General Data Protection Regulation 2016/679 (GDPR), Article 6(1)(e) and Article 9(2)(j); Data Protection Act, Sections 4 and 6).

### 2.2. Outcome Variables

Information on infant birth weight and length were obtained from the National Birth Register of Finland. Birth measure z-scores were calculated using the recently published references specific to the Finnish population [26]. Perinatal characteristics also included child sex, duration of gestation, and if the infant was born preterm (occurring before <37 weeks’ gestation) and post-term (occurring ≥ 42 weeks’ gestation). One infant was born severely premature at 27 weeks and was excluded from the analysis as due to being significantly different from others.

### 2.3. Paternal Variables: BMI and Index of Diet Quality (IDQ)

Paternal self-reported height and weight were collected from self-administered questionnaires upon recruitment in late pregnancy (30 weeks) for calculation of paternal BMI (kg/m^2^).

Paternal diet quality was examined in late pregnancy (30 weeks’ gestation) using the Index of Diet Quality (IDQ), which measures adherence to health-promoting diet and nutrition recommendations [27]. Briefly, this included the consumption of key nutrients; saturated fatty acids, the ratio of unsaturated versus saturated fatty acids, protein, saccharose, fibre, calcium, iron and vitamin C, which have been associated with health and lower risk of disease in several studies [24]. The IDQ score used in the statistical models was the continuous variable, and was categorised as seen in Table 1, by setting the statistically defined cutoff value at 10, with scores below 10 points indicating unhealthy diets and non-adherence and scores of 10–15 points indicating a health-promoting diet and adherence dietary guidelines. Two fathers were excluded from the analyses due to being extreme outliers.

### 2.4. Background Variables

#### 2.4.1. Paternal Age, Education, and Smoking

Information regarding paternal age, education (basic/advanced), and self-reported smoking habits (yes/no) were obtained from the questionnaires administered during pregnancy. Education was classified into two categories: advanced education or low education based on the highest education attained. Those who had studied at a University of Applied Sciences or higher were classified as “advanced”. The advanced level included any academic degree (bachelor’s, master’s, licentiate, or doctoral degree). University of Applied Sciences are practice-oriented, with the goal of educating students for professional work life compared with traditional universities, which are research-driven. Those who had no professional training or a maximum of an intermediate level of vocational training were classified as “basic”.

#### 2.4.2. Covariate Variables: Maternal Variables

Maternal age, self-reported smoking habits (yes/no), education (basic/advanced), and self-reported height and weight were collected from self-administered questionnaires upon recruitment for calculation of pre-pregnancy BMI (kg/m^2^). Maternal primiparity (bearing a child for the first time) and gestational diabetes mellitus (GDM) was obtained from Medical Birth Registers. Maternal diet quality was examined using the Index of Diet Quality (IDQ) in late pregnancy and classified similar to maternal as father IDQ. The continuous variable was used in the models.

### 2.5. Statistical Analyses

All statistical analyses were performed using SPSS statistical software package version 27 (IBM SPSS Statistics for Windows, Version 27.0. IBM Corp., Armonk, NY, USA). First, basic characteristics of participants were described. Paired *t*-tests were carried out to examine the differences between mothers and fathers in their characteristics and independent *t*-tests to examine the differences between male and female offspring. Outcome variables, birth weight z-score, and birth length z-score were assessed for normality and were found to be normally distributed. Extreme outliers were removed if more than 3 SD over the mean. To investigate associations between offspring birth measures and paternal BMI and IDQ, multiple linear regression analyses (GLMs) were carried out for birth weight z-score and birth length z-scores separately as dependent variables. Both birth weight and length z-score models were adjusted for Maternal height, Maternal BMI, Pre-pregnancy smoker, Maternal diet quality score, Primiparity, and GD (Yes/No). Maternal education was also added to the model due to previous studies showing significant associations between maternal education and offspring birth measures [28,29,30]. In addition to paternal BMI and IDQ, which were of main interest in the models, other paternal variables considered potentially relevant to birth weight and length z-scores included paternal age, paternal height, and low paternal education, and they were also included in the models. For the paternal diet quality, the continuous variable of paternal diet was used. Furthermore, interactions between paternal BMI and IDQ were tested to investigate if paternal weight was differently associated with birth weight and length z-scores depending on the father’s diet. Firstly, paternal BMI and IDQ were checked and only showed a moderate level of multicollinearity and therefore could be included in the same analysis. To reduce collinearity in the models with the interactions, paternal BMI values were mean-centered and paternal diet was included as a categorical IDQ variable [31]. Lastly, the data was analysed for any sex-specific effects in the offspring.

## 3. Results

### 3.1. Study Characteristics

Characteristics of fathers (*n* = 1640), mothers (*n* = 1640), and the infants (*n* = 1640) participating in the STEPS study are presented in Table 1 as Mean ± SD or expressed as Percentage (%). The mean age of fathers was 32.9 years (±5.4) and 30.8 years (±4.6) for mothers. The mean BMI of fathers was 26 (±3.5) (Table 1). Fathers had a poorer diet score (8.9 ± 2.3) compared with mothers (10.2 ± 2.1), with 59.8% of fathers being classed as having a “bad” diet at 30 weeks of gestation compared to 33.5% of mothers (Table 1). Less than 10% of mothers in the cohort had gestational diabetes, and in 53.7% of the cohort, the offspring was the first born (Table 1). Table 2 presents the characteristics of the offspring in this study. Only a small number of children were born pre-term (2.7%), and the mean gestational age for boys and girls was 39.9 weeks (±1.5). The mean birthweight z-score of boys was −0.03 (±1.1) and −0.07 (±1.2) for girls. The mean birth length z-score for boys was 0.07 (±1.1) for girls and 0.03 (±1.1).

### 3.2. Paternal BMI, Diet, and Offspring Birth Measures

Results examining associations between paternal BMI and diet on offspring birthweight and birth length z-scores are presented in Table 3. In adjusted models, paternal dietary quality score was not associated with increased birth length (β = 0.00, *p* = 0.774) or birth weight z-scores (β = −0.01, *p* = 0.515). Paternal BMI was not associated with birth length (β = 0.07, *p* = 0.291) or birth weight z-scores (β = 0.00, *p* = 0.884). Interactions between paternal diet score and paternal BMI are presented in Table 4. There were no sex-specific differences observed for any outcomes measured.

## 4. Discussion

To the best of our knowledge, this is the first study to assess the relationship between paternal diet quality and offspring birth measures in a Finnish cohort. We found that neither birth weight nor birth length z-scores were significantly influenced by paternal diet quality score or BMI even when adjusted for maternal and other paternal factors. Most fathers in this study (59.8%) were classed as having an “unhealthy” diet determined by a diet quality score of below 10, and the mean paternal BMI was 26 ± 3.5, which falls into the overweight BMI category.

Our results suggest maternal rather than paternal factors may be more important in determining final neonatal size. Nonetheless it remains important to understand the paternal contribution of early exposures on offspring health, which is often overlooked in human studies. Understanding the paternal, in addition to the maternal, influence will only benefit human health and future research, particularly in the context of tackling the steadily increasing malnutrition prevalence worldwide, with approximately 150 million children under 5 estimated to be stunted, 45 million estimated to be wasted, and 37 million children overweight or living with obesity [32].

Although our results reflect no significant associations between paternal diet and BMI and infant birth measures, it is now widely accepted that paternal exposure to environmental factors, particularly a nutritionally unbalanced diet, can alter the development of the offspring and are observed both intergenerationally and transgenerationally [33]. Yet, while maternal BMI and diet is widely looked at, there are limited studies which have looked at paternal BMI and diet and effects on offspring birth measures. For example, evidence in the literature suggests that paternal methyl donor intake is critical for offspring growth and development [34]. It is well-known that methyl donor nutrients, including folate, methionine, choline, and certain B vitamins, play a relevant role in DNA methylation by acting as methyl donors and may impact the health of the offspring [34]. Studies have shown low paternal dietary folate in mice leads to craniofacial and musculoskeletal birth defects in offspring, in addition to altered sperm DNA methylation [35]. Intake of these nutrients are through consumption of foods such as fruits and green leafy vegetables, beans and legumes, nuts, and fortified foods, all of which are obtained through a well-balanced diet and adherence to food-based dietary guidelines. It may be plausible that fathers who have better dietary quality are consuming these key methyl donor nutrients and contribute to offspring development and prevent impaired growth outcomes, including stunting. For instance, a recent study showed that sperm of males consuming unhealthy foods, or food items high in saturated fatty acids, such as fried foods, were more methylated and had reduced sperm motility compared to that of males consuming vegetables, fruits, and nuts [36], showing the potential negative epigenetic impact of unhealthy eating behaviours. However, our analysis was carried out on paternal diet in late gestation (30 weeks), which might not be a very good proxy measure for preconception paternal diet. Hence, while we did not find a significant association between paternal diet and birth measures, we recommend that future research should explore the paternal diet at the pre-conception time point, to accurately determine pre-conception influences on offspring growth.

Birth measures, including weight and length, are important to study as they have shown to be a cause of morbidity and mortality in infants and children. This is particularly true for children that have a low length for age [37] or stunting. The WHO definition for stunting is low length for age < 45 cm [38]. Stunting has severe health consequences, including poor cognition and education performance, and increased risk of nutrition-related chronic diseases in combination with excessive childhood weight gain in later years [38]. Among the STEPS participants, 3.7% of neonates met the criteria for low length for age. On the other hand, the global prevalence of low birth weight, defined as neonates weighing < 2500 g, is estimated to be 15–20% and is most apparent in low- and middle-income countries [39] and lowest in the Nordic countries including Finland, with less than 5% of live births defined as low birth weight [40]. This low prevalence is reflected in this present study, with an average birth weight of 3548.0 g and 2.3% of the cohort being classed as low birth weight. Hence, the fact that our study was carried out in a high-income and Nordic country might have affected our findings. In fact, it is possible that paternal BMI and diet might be linked to more pronounced differences in birth measures in low- and middle-income countries, where food security and stunting are significant problems [41,42].

While most studies looking at parental influences on offspring outcomes examine birth weight outcomes, our study also examined birth length, which in contrast to birth weight has not been studied widely and it may be useful to study these two birth measures independently. Although birth length is largely genetically determined, it has been previously shown that external factors, including parental smoking and maternal nutrient intake, contribute to decreased birth length [43,44]. Furthermore, birth length is considered a better predictor of adult height and weight than birth weight and should be considered a risk factor for morbidity in adulthood, as short stature in adulthood has been associated with increased health risks, including cardiovascular disease [45]. In fact, intrauterine weight and length growth have been shown to have differing periods of major growth during pregnancy, with peak velocity of length growth observed at 16 weeks’ gestation compared to peak velocity of weight at approximately 33 weeks’ gestation [46]. Therefore, restriction in uterine growth can follow two patterns: those with decreased length and weight and those with normal length but decreased weight. Growth restriction occurring in the earlier trimesters are associated with maternal chronic malnutrition, smoking, and genetic factors [46]. Indeed, maternal malnutrition has been associated with poor birth outcomes, including shorter birth length [47]. Yet, a growing body of evidence, mainly gathered from animal studies, suggests paternal undernutrition before conception can negatively affect sperm quality, alter epigenetic profiles in spermatozoa, and increase the risk of reduced postnatal weight and growth in the offspring while also negatively impacting their metabolic health in adulthood [48]. Nonetheless, there is a paucity of literature analysing birth length in human studies, particularly in relation to paternal factors. The few studies carried out in this context focused on paternal anthropometric measures and found that paternal height is positively associated with offspring birth length [22,49], while paternal weight is typically not associated with birth length [12,49]. This is a finding that was also observed in our study. Interestingly, additional studies showed positive associations between paternal anthropometric measures and infant measures at birth and in later childhood [50,51]. This suggests that exploring both paternal and maternal contribution to infant birth length should be included in future analyses given the evidence surrounding birth length as a predictor for adult height and therefore the associated morbidities in later life, as mentioned previously.

It is worth noting that while this study did not find significant associations between paternal diet and BMI and infant birth weight and length, significant associations may be observed later in childhood. Studies have shown positive associations between paternal pre-conception lifestyle factors, including smoking and childhood obesity, at 5 years of age [52], and paternal obesity and increased risk of offspring obesity during adolescence and adulthood [53,54]. Research has shown that early exposures in utero, including epigenetic changes, invoke perturbations during critical periods of the development of a phenotype triggered by insults such as nutrition, stress, toxins, prior to conception, during embryonic and fetal life, perinatally, and across early stages of growth and development [55,56]. These early insults may lead to disruptions in organ growth, differentiation and function, immune response, metabolic regulation, and preferences and behaviours in adulthood [56]. Therefore, investigating follow-up data in later childhood in this cohort might provide deeper insights into paternal influences on their offspring’s growth and development, particularly given that childhood obesity is one of the most serious challenges globally today with profound and wide-ranging impacts on an individual’s health [57].

Strengths of the present study include the novelty of the topic; to our knowledge, this is the first study to examine paternal diet quality in addition to BMI with their offspring’s birth measures in humans. Additionally, the study included collection of detailed paternal data such as age, smoking, and education status, and therefore provided the opportunity to account for many confounding factors during the analysis. Our study also had a reasonable sample size of fathers included in this study for analyses. Although the sample was relatively small compared with some large cross-sectional studies, other similarly sized familial studies have been carried out that reported significant paternal as well as maternal influences on offspring outcomes [58,59].

Limitations of the study included the use of self-reported anthropometric measures to calculate the BMI and self-reported dietary data. These may have introduced bias, as respondents may have systematically under- or over-reported their body measurements and/or their food consumption. However, the mean BMI is reflective of the average BMI of 25.7 at time of this data collection [60]. However it must also be noted that since 2017, the average BMI has increased by 2 kg/m^2^, to 27.7 [60], and future studies should include more recent data to assess paternal BMI influences on offspring outcomes, reflective of a population with an increased BMI. Nonetheless, self-reported weight and height are often used in large epidemiologic studies owing to the feasibility and cost, with measured values of self-reported weight tending to be underestimated and self-reported height overestimated [61,62,63]. It has been reported in many studies that people living with overweight or obesity under-report their weight more those who are classed as having a normal weight [64,65].

Additional weaknesses include the fact that dietary information was only collected for 208 participants at 30 weeks’ gestation. In relation to the fewer respondents to dietary information, there was no difference in variables such as BMI, age, or height among fathers who had dietary information collected and those who did not. Moreover, there is a paucity in the literature examining weight patterns of expectant fathers during pregnancy. A longitudinal study on 10,000 men by Garfield et al. showed entrance into fatherhood predicted increased body mass index (BMI), whereas non-fathers showed decreasing BMIs over the same time period [66]. The mechanisms are unclear. However, one study suggests male monkeys increased their weight by 20% during pregnancy compared with control non-expectant males, a phenomenon known as couvade syndrome, which may be a biological advantage in preparing for an increase in expectant energetic expenditure once the offspring arrives [67]. Therefore, our findings cannot be defined as paternal pre-conception dietary and BMI information. Lastly, there was also no test for biological paternity. Hence, we could only assume that the self-reported father is the biological father of the infant.

## 5. Conclusions

Our findings showed that paternal BMI and diet quality were not significantly associated with offspring birth weight or length z-scores when controlled for maternal or paternal factors. The long-term consequences of paternal dietary influence on offspring anthropometric measurements require follow-up as evidence, largely from animal studies that show negative associations between paternal diet and BMI and offspring outcomes, which may relate to the quality in sperm or seminal plasma and warrants further investigation in human studies. A better understanding of paternal influences on offspring growth and development, including health outcomes, could have important implications for public health. It is hypothesised that there are many possible mechanisms through which paternal exposures might influence offspring health and development. The importance of maternal lifestyle and diet preconception and during pregnancy is well-recognized. However, there is currently a lack of awareness regarding the father’s contribution to offspring health. Continued research to include both parents will be informative to the field of DOHaD and may guide public health policies in the future.

## Figures and Tables

**Table 1:** Parental Study Characteristics. Statistical differences between mothers and fathers were tested with *t*-tests.

	Paternal or Fathers (*n* = 1640)	Maternal or Mothers (*n* = 1640)	*p* Values
Variable			
Age, years	32.9 (5.4)	30.8 (4.6)	<0.001
BMI, kg/m^2^	26.0 (3.5)	24.3 (4.9)	<0.001
Primiparity n (%)	--	878 (53.7)	
Smoker n (%)	203 (20.3)	182 (16.6)	<0.001
Smoker during pregnancy (%)		46 (4.2)	
Education (Advanced) n (%)	992 (65.1)	1206 (78.4)	<0.001
Family Income (>€3000) n (%)	878 (54.0)		
Gestational diabetes n (%)	--	142 (8.6)	
IDQ score, mean (SD)	8.9 (2.3)	10.2 (2.1)	<0.001
Diet quality n (%)			<0.001
Healthy	332 (40.2)	669 (66.5)	
Unhealthy	494 (59.8)	337 (33.5)	

Significance *p* < 0.05 observed by paired samples *t*-test.

**Table 2:** Offspring Study Characteristics. Statistical differences between boys and girls were tested with *t*-tests.

Variable	Total (*n* = 1640)	Boys (*n* = 863)	Girls (*n* = 777)	*p* Value
Duration of gestation (weeks)	39.9 (1.5)	39.9 (1.5)	39.9 (1.5)	0.998
Birth categories n (%)			0.001	
Preterm (<37 gw)	34 (2.1)	13 (1.5)	21 (2.7)	
Term (37–42 gw)	1575 (97.9)	832 (98.5)	743 (97.3)	
Mode of delivery, caesarean section	204 (12.5)	104 (12.1)	100 (12.9)	0.983
Birth length, cm	50.9 (2.1)	51.3 (2.1)	50.4 (2.1)	0.768
Birth length, z-score	0.05 (1.1)	0.07 (1.1)	0.03 (1.1)	0.147
Birth weight, g	3549.5 (499.1)	3609.4 (495.3)	3483.1 (495.2)	0.831
Birth weight, z-score	−0.05 (1.1)	−0.03 (1.1)	−0.07(1.2)	0.221

Significance *p* < 0.05 observed by independent sample *t*-test.

**Table 3:** GLM results of associations between Paternal BMI, Paternal Diet Quality Score, and offspring birth measures.

	Total Offspring Birth Length z-Score (*n* = 1579)	Boys Birth Length z-Score (*n* = 834)	Girls Birth Length z-Score (*n* = 745)	Total Birth Weight z-Score (*n* = 1633)	Boys Birth Weight z-Score (*n* = 859)	Girls Birth Weight z-Score (*n* = 774)
	B	95% C.I	*p*-Value	B	95% C.I	*p*-Value
Paternal BMI	0.00	−0.02–0.03	0.774	−0.01	0.02−0.03	0.964
Paternal Diet Quality Score	0.07	−0.02–0.06	0.291	0.02	0.03−0.03	0.436

Each model was adjusted for Offspring Gender, Paternal age, Maternal age, Paternal Height, Maternal height, Paternal height, Maternal BMI, Maternal Diet quality Score, Primiparity, Maternal education level (Advanced/Basic), Paternal education level (Advanced/Basic), Maternal Gestational diabetes (Yes/No), and Paternal Smoking status (Yes/No). Significance *p* < 0.05.

**Table 4:** Test of Model Effects: Associations between Paternal BMI (mean-centered) * Paternal Diet Class Interaction.

	Total Offspring Birth Length z-Score (*n* = 1579)	Boys Birth Length z-Score (*n* = 834)	Girls Birth Length z-Score (*n* = 745)	Total Birth Weight z-Score (*n* = 1633)	Boys Birth Weight z-Score (*n* = 859)	Girls Birth Weight z-Score (*n* = 774)
Interaction						
Paternal BMI * Paternal Diet Class	Wald–Chi Square	*p*-value	Wald–Chi Square	*p*-value	Wald–Chi Square	*p*-value
Main Effects	0.028	0.866	0.117	0.733	0.003	0.954
Paternal Diet Class						

Significance *p* < 0.05.

## Data Availability

Data are unavailable due to privacy or ethical restrictions. Requests to access the data sets should be directed to hanna.lagstrom@utu.fi.
